# The Utility of Therapeutic Anticoagulation in the Perioperative Period in Patients Presenting in Emergency Surgical Department With Extremity Vascular Injuries

**DOI:** 10.7759/cureus.8473

**Published:** 2020-06-06

**Authors:** Ayesha Masood, Nuaman A Danawar, Andrew Mekaiel, Sumit Raut, Bilal Haider Malik

**Affiliations:** 1 General Surgery, California Institute of Behavioral Neurosciences and Psychology, Fairfield, USA; 2 Internal Medicine, California Institute of Behavioral Neurosciences and Psychology, Fairfield, USA

**Keywords:** vascular injuries, vascular system injuries and anticoagulants, extremity vascular trauma, systemic anticoagulation, intraoperative/postoperative anticoagulation, emergency vascular injuries, extremity vascular repair

## Abstract

Extremity vascular trauma is a challenging surgical emergency in both civilian population and combat environment. It requires vigilant diagnosis and prompt treatment to minimize limb loss and mortality. A multidisciplinary team approach is required to deal with shock states, concomitant abdominal injuries, head injuries, and fractures with significant tissue loss and psychological stress.

Anticoagulation is frequently used during traumatic vascular repair to avoid repair site thrombosis, postoperative deep venous thrombosis, and pulmonary embolism (PE). In this review article, we are going to search about how frequent is the use of anticoagulation in terms of limb salvage rates, and mortality rates or side effects of anticoagulation in terms of risk of bleeding episodes, and the need for future prospective studies.

Extremity vascular trauma is managed by a variety of methods including open repairs, endovascular repairs, and nonoperative management. Most of the literature demonstrates the use of systemic or regional anticoagulation in the management of vascular injuries with the improvement in limb salvage rates and reduced morbidities but confounding factors lead to variable results. Some studies show an increased risk of bleeding in trauma patients with the use of anticoagulants in trauma settings without any significant effect on repair site thrombosis. More comprehensive studies and randomized controlled trials are needed to confirm the importance of perioperative anticoagulation while avoiding the confounding factors in terms of injury severity scores, ischemia time, demographics of patients, modes of injury, comorbidities, grades of shock, concomitant injuries that need anticoagulation like venous injuries or intracranial injuries that are contraindications to the use of anticoagulation, type of anticoagulation and expertise available as well as the experience level of the operating surgeon. Literature also reveals the use of new oral anticoagulants (e.g., dabigatran) to be associated with lesser bleeding episodes when compared to warfarin, so in future, we can check the feasibility of these agents to reduce the bleeding episodes and at the same time improve the limb salvage rates.

## Introduction and background

Extremity vascular trauma is a major cause of morbidity and mortality leading to limb loss or life loss. It results in significant physical, psychological, social, and personal problems for patients and their families. Amputation rates vary according to the risk factors associated with an injury like associated soft tissue damage, bone damage and degree of shock measured by injury scoring systems, that is, mangled extremity severity score (MESS) and injury severity score (ISS), associated orthopedic fixation, and use of systemic anticoagulation [1,2]. Extremity vascular trauma has varied etiology including road traffic accidents, firearm injuries, stab wounds, and suicidal attempts [3]. Patients presenting to the emergency department with traumatic vascular injuries dealt by trauma surgeons compared with vascular surgeons show similar outcomes in selected patients [4].

Historically arterial injuries were ligated, but then there was a significant change in the management of vascular injuries from ligation towards repair as a result of the Korean War with improvement in amputation rates from 51% to 13% [5]. Popliteal artery injury was associated with the highest amputation rate of 34.2% [5].

Mortality rates vary from 5% to 10% depending on the vessels injured [1]. Amputation rates for isolated lower extremity injuries are 6.5% reported by Kauvar and colleagues, who collected data from the national trauma database (NTDB) from January 2002 to December 2006 [6].

Associated with arterial injuries are fractures, mangled extremities, head injuries, thoracoabdominal injuries, and combined arterial and venous injuries [3,7].

Usual methods of repair are debridement with end-to-end anastomosis, reversed venous interposition grafts, synthetic grafts, and endovascular repair if available. Venous injuries are either ligated or repaired [1]. But if the patient is unstable and unable to withstand a lengthy procedure, management is shifted towards intravascular shunting or ligation [1]. While the patients with severe injuries and mangled extremities who would not benefit from limb salvage are recommended primary amputation to avoid myonecrosis leading to acute renal failure and loss of life (limb saving vs life-saving strategies) [8].

According to previous literature, systemic anticoagulation is given at the time of repair in the absence of any contraindications to anticoagulation. Standard doses are used in isolated vascular injuries while these doses are adjusted according to the associated injuries and in case of contraindications to their use, only a regional heparin solution is used while its use can result in an increased risk of hemorrhage in trauma settings [1,9].

It has been reported that the use of systemic anticoagulation leads to lower rates of limb loss and anastomosis site thrombosis by increasing the patency of microvasculature, which has a major impact on the life of patients in terms of long-term morbidities [2,8,10].

In this review article, we are going to search about how frequent is the use of systemic anticoagulation and its impact on the prevention of limb loss because of failed vascular anastomosis and thrombosis in comparison with its avoidance due to bleeding episodes and revisits to the operation theatre. It will guide us in the future about the use of anticoagulation in vascular trauma settings and in conducting new experimental studies to confirm the importance of anticoagulation in vascular extremity trauma and its utility in saving limbs and effects on mortality.

## Review

Spectrum of extremity vascular injuries

Extremity vascular injuries after trauma are an important surgical challenge for trauma surgeons, vascular surgeons, and patients at the same time. Extremity vascular trauma has multiple causes and treatment options. It has varied etiology being motor vehicle accidents, street crimes, explosions, industrial accidents, and most common being penetrating trauma with increasing trends towards penetrating trauma by gunshot wounds [11-13]. Extremity vascular injuries among overall vascular trauma patients range from 30 to 45% to 50%[7,14].Penetrating trauma accounts for 64% to 82% cases [14-16]. Upper extremity involved in 30%, more in civilian trauma, and lower extremity more in military trauma [15,17]. Gunshot wounds range from 15% to 45% in incidence and stab wounds are 55% to 65% [7]. Mortality is 2.8% with penetrating vascular trauma, mostly because of more proximal vascular injuries [7]. In another study, mortality is 5.4% associated with more than eight hours of presentation along with disseminated intravascular coagulation (DIC) being an important cause [11]. The most common mode of injury reported is occlusion or transaction and, in rare cases, vasospasm [7,14]. Most commonly injured vessels in lower extremity include superficial femoral artery, second most common is the popliteal artery and tibial artery, and in the upper extremity, it includes brachial artery associated with a median nerve injury in 15% cases while subclavian or axillary arterial injuries are less common due to their protected position and often misdiagnosed because of subtle findings on examination [11,14,18,19,20]. Patients with complete brachial plexus injury and critical hand ischemia may better be treated with primary above elbow amputation rather than to adopt complex repair with futile results as if even limb is saved but still chronic debilitating pain and motor impairment compel towards amputation, but the decision is difficult. More distal injuries have better outcomes [15]. 3.4% of cases have combined injury on either side of the knee and 12.3% of cases have more than one arterial injuries [14,18]. Concomitant injuries include mostly bone and veins [11,14,18]. Associated injuries to abdomen, chest, head and neck are 15.4% and 12.5% [14,20]. The mean age of presentation is 30 to 40 years and male gender [11,14,15,16,18,19,20]. Injuries to the deep femoral artery and crural artery are not limb-threatening [18]. The mean time to injury and hospital admission is 2 hours, 8 hours, or 5.5 hours according to different studies [11,16,18]. Popliteal artery injuries are difficult to manage [21]. No valid anticoagulation protocol exists for extremity injuries [22,23]. Blunt trauma is the second most common cause after penetrating injuries being 33% [14]. Temporary intravascular shunts are useful in damage control surgeries in order to stabilize the patient before definitive repair [24].

Extremity vascular trauma has varied mechanisms most common being penetrating trauma by gunshot injuries and most commonly injured vessels being popliteal artery and brachial artery. Most of the patients are young males. Prognosis of upper extremity vascular injury is better than lower extremity while lower extremity injuries are increasing with time. Most concomitant injuries involve bones, veins and nerves associated with worse outcomes. Associated injuries also occur. Mortality rates vary from 2.8% to 5.6% associated with DIC, shock and polytrauma. Long-term morbidity is not followed by most studies. Kim et al. studied 24 patients in a retrospective study for risk factors for amputation [25]. Franz et al., in a retrospective review article, studied 66 patients with lower limb vascular injury, in which they took approval from the ethical committee, involved a multidisciplinary team of orthopedic surgeons, vascular surgeons and plastic surgeons [14]. Both studies showed a preponderance of male patients. According to Kim et al., popliteal artery injury was more prevalent resulting from blunt trauma, while it was tibial and superficial femoral artery due to penetrating trauma according to Franz et al [14,25]. Kim et al. described a fasciotomy rate of 20.8% while it was 38.2% in Franz et al. study [14,25]. Kim et al. described amputation rates of 20.8% due to failed revascularization, soft tissue injury and osteomyelitis [25]. Franz et al. described endovascular repairs also, done in 6.8% cases without any complications and overall limb salvage rate was 95.1% due to advances in repair techniques and decreasing the ischemia time along with the liberal use of fasciotomy [14]. Also, 10% of cases of nonocclusive injuries were managed nonoperatively with aspirin or clopidogrel and successfully cured. Long-term follow-up is lacking in these studies.

 

Strategies used to deal with the challenge

Traumatic extremity vascular injuries are managed by an array of procedures and also managed conservatively in some situations. Management starts with resuscitation with clinical examination and direct exploration or investigations followed by repairs or medical management or amputations to concomitant fasciotomies, vein repairs/ligations, fracture fixations, nerve repairs/tagging, and intraoperative or postoperative anticoagulation to discharge in stable condition or with secondary amputation or resulting in loss of life. Patients presenting with the lower extremity vascular trauma resuscitated and evaluated. Those with hard signs of vascular injury like absent distal pulses, bruit/thrill, expanding hematoma were taken directly to the operation room for exploration. Those with soft signs of injury such as feeble pulses, hematoma near the major vessel, were examined, those with ABI <1 underwent duplex scan or CT angiography and if there was an injury to the vessel, then these patients underwent revascularization. The most common method of repair was either end-to-end anastomosis or reversed saphenous interposition graft with a saphenous vein as the most used conduit for common femoral and popliteal arteries and in some cases, polytetrafluoroethylene (PTFE) prosthetic grafts were also used. Tibial artery injuries were ligated [14]. Some patients with isolated profunda femoris artery or crural artery injury underwent coil embolization [14,18]. Patients with upper extremity injuries were also repaired in a similar way whenever possible, while radial artery injuries were ligated in many cases with a successful outcome [11,15]. Intravascular shunts were used to buy time in unstable patients and delayed repair was done after stabilizing the patient and in some studies, shunts were not used [11,14,18]. Arterial repairs took precedence over other associated injuries such as fractures (except isolated knee dislocation causing ischemia and unstable tibial fractures), nerve injuries, and soft tissue damage to save limb ischemia time and vascular repair integrity was reassessed by the vascular surgeon at the end of procedure [18]. Completion angiograms routinely were done and if there was technical difficulty then revised after a multidisciplinary meeting [18]. Patients with extremely mangled, insensate, and gangrenous limbs were amputated before any limb salvage. Repair surveillance was done in the postoperative period and if there were signs of ischemia then decisions made after a multidisciplinary meeting (MDM). Fasciotomy was a crucial component of management with the absolute indication being tense compartment. Initial soft tissue cover was provided by split skin grafts and delayed flaps done after eradication of infection [14,11]. Medical management pursued in nonocclusive injuries with clopidogrel 75 mg with aspirin 81 mg per day or aspirin 81 mg alone if there was any contraindication to clopidogrel [7,14] in cases of <5 mm intimal disruption, adherent intimal flaps, intact distal circulation, no active bleeding, and vasospasm. These patients were managed with duplex scans, ankle brachial indexes and CT angiography (CTA), if showing signs of ischemia then proceeded with the surgery. It resulted in 10% of the false aneurysm that was treated successfully [14].

Patient management starts with resuscitation and expeditious yet careful clinical evaluation and duplex scan if time allows, for hard signs of ischemia, which if present, mandate emergent exploration. An MDM is an integral component of management and repair needs an experienced surgeon. Soft signs of ischemia undergo CTA and managed accordingly, maybe expectantly but under vigilant surveillance. The most common methods of repair are vein interposition grafts or end-to-end repairs and with advances in endovascular repair also showing promise in simpler lesions. Fasciotomy is the crucial part of revascularization and an important predictor of amputation. Intraoperative shunts may be used if a patient is unstable and expertise is not available but should be kept for a minimum period. Cakir et al. discussed the management of patients with concomitant vascular injuries with fractures in a retrospective review of 192 patients [8]. Musonza et al. described a case report of a patient with bilateral popliteal artery injury along with concomitant fractures of right tibia fibula and left knee joint and managed the patient without the need of amputation though at the end there was sensory impairment in right leg and extension of the left knee [21]. Cakir et al. in their study did mention the use of early fasciotomy associated with improved limb salvage rate and routine use of intraoperative systemic anticoagulation as well as they repaired vessel before orthopedic intervention [8]. Musonza et al. described the use of prophylactic fasciotomy in crushed, concomitant venous injuries and ischemic limbs of more than six-hour duration as well as they also used systemic anticoagulation [21]. Antibiotic beads were used in the treatment of fractures to prevent osteomyelitis. The use of shunts was emphasized to buy time for repair. Tourniquets or zone 3 resuscitative endovascular balloon occlusion devices (REBOA) are required for hemorrhage control but as it was a case report so larger studies are needed to prove the fact. Chronic infections and causalgia are present as chronic morbidities in salvaged limbs.

Anticoagulation in perioperative period

The use of anticoagulation in extremity vascular trauma is a debatable aspect with varying suggestions and experiences. Its impact on microvascular thrombosis leading to graft failure, secondary amputation and postoperative Deep venous thrombosis (DVT) is established by some studies and textbooks, while in other studies, there is no role of anticoagulation. Goerlich et al., in a case report, described the use of single antiplatelet therapy in a patient with gunshot wound leading to arterial vasospasm who was successfully managed expectantly with vigilant monitoring [7]. Hafez et al. described only flushing of vessel distally after thrombectomy before vessel repair with heparinized saline solution (5,000 U/L) without the routine use of systemic anticoagulation and showed that graft occlusion was the leading cause of failure of revascularization leading to amputation in their results but it was comparable to the amputation rate of study by Wagner et al who administered full doses of heparin to their patients [2,18]. Franz et al. reviewed a cohort of 65 patients who were given antiplatelet therapy in nonocclusive injuries, which were managed expectantly and no amputation resulted [14]. Khan et al. described the use of low molecular weight heparin (LMWH) in the postoperative period for DVT prophylaxis till the patients were out of bed with a limb salvage rate of 41% and graft thrombosis being 6.2% with the main cause of limb loss being delayed presentation >8 hours [11]. Fitridge et al. in their 23-year period of 114 patients with upper extremity injuries used intraoperative 5,000 units of heparin unless contraindicated with no complication seen with use while poor outcomes were associated with complete brachial plexus injuries [15]. Klocker et al. described 152 patients with vein grafts for upper and lower extremity injuries and limb loss was significantly associated with graft occlusion being 9.9% [20]. Full systemic anticoagulation with unfractionated heparin was given as 5,000 U bolus and continuous infusion perioperatively for 24 hours with aspirin for three months 100 mg per day or life long if other comorbid associated. But it could not be well evaluated whether it was associated with soft tissue injuries, anticoagulation or technical failures. Long-term follow-up was six years and two patients developed occlusion managed conservatively and one patient developed dilatation treated with graft replacement [20]. Musonza et al. described anticoagulation to be given after hemorrhage control in their case report of bilateral popliteal artery trauma [21]. di Silva et al. described thrombectomy of proximal and distal segments and heparinized saline flushing for the distal end and defined two amputations, one from sepsis and other from graft failure and also described that ischemia time is not the absolute factor to defer revascularization [16]. Lebowitz and Matzon described that despite the common problem of arterial thrombosis, the standard anticoagulation regimen is yet to be defined [22]. Cakir et al. described the use of systemic anticoagulation for three days in 192 patients with both limb injuries, unless contraindicated and amputation was associated with extensive tissue damage [8]. Humphries et al. described that the use of systemic anticoagulation did not result in lowering the repair failure or limb loss and there were no adverse outcomes of anticoagulation as well [9]. Woodward et al. described 488 vascular trauma injuries with regional use of heparin [23]. Limb loss was associated with early repair failure in 7% cases and soft tissue damage in 0.7% cases [23]. Patients with femoropopliteal injuries had systemic anticoagulation unless contraindicated with a varied rate of amputation including failed repair [24]. Ali Yousef et al. described the insignificant effect of heparin in the prevention of microvascular surgery and be better by antiplatelet agents in femoral vessels in 20 rats experimentally [26]. Lang et al. described the use of regional anticoagulation at the time of vascular repair with the amputation rate of 28% in 64 patients and was mostly due to vascular repair failure [27]. Liang et al. described the use of antiplatelet therapy as aspirin in nonoperative cases only and used either systemic (100 u/kg) or regional heparinization (12,500 units/250 mL normal saline or 50 units/mL with an injection of 15-20 mL/end of vessel) but the outcome was unclear [28]. Patients should be put on continuous anticoagulation if not contraindicated to reduce repair failure leading to limb loss in upper extremities [29]. Popliteal artery repairs were done after flushing with hepsal solution but limb salvage correlation was not described [30]. Melton et al. described that intraoperative use of heparin or local urokinase or both was directly associated with improved limb salvage rates [31]. It was studied that the use of systemic anticoagulation and extra-anatomic vein bypass of popliteal artery repair leads to more successful outcomes with a 91% limb salvage rate suggesting routine use of systemic anticoagulation without any complications of bleeding [32]. Patients with blunt vascular trauma of head and neck have serious morbidities and anticoagulation is used but verification needs randomized controlled trials (RCTs) [33]. Anticoagulation in upper extremity arterial injury repairs should be given unless contraindicated [34]. Intraoperative use of anticoagulation is associated with lower-limb loss without any significant bleeding complications [35]. Although regional hepsal solution is used, perioperative use is not well demonstrated [36]. In a study of 1,524 patients, it was noted that there is no role of anticoagulation to prevent thrombosis although there is no significant bleeding risk as well [37]. In a multicenter prospective cohort study of 193 patients, it was observed that intraoperative systemic anticoagulation was not associated with improvement of limb salvage or vascular thrombosis but resulted in increased use of blood products [38]. Maher et al. described in a retrospective cohort study of 323 patients, improvement of limb patency rates with the use of intraoperative systemic anticoagulation [10]. Otuki et al. in their study of the effect of direct oral anticoagulants in comparison to warfarin in terms of bleeding risk in already anticoagulated patients undergoing catheter ablations for cardiac arrhythmias showed that the level of protein C, S and thrombin was not affected by these new oral anticoagulants resulting in lesser risk of bleeding episodes [39]. However, there was bias in the selection of patients and treatments already taken by the patients [39].

**Figure 1:**
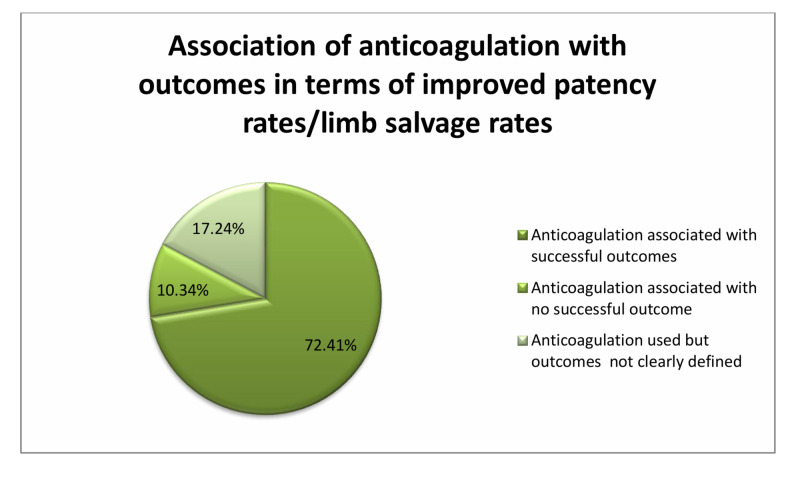
Studies showing the use of anticoagulation with variable outcomes

Among the 29 studies discussed above, 21 (72.41%) studies demonstrate the use of anticoagulation either as regional or systemic therapy associated with significant limb salvage rates/improved patency of anastomosis. Three studies (10.34%) showed no benefit of anticoagulation of which, in one study it was associated with increased bleeding episodes and use of blood products, while in two studies there was no rise in bleeding risk. In five studies (17.24%) anticoagulation was used but no clear benefit was defined (Figure 1; Table 1).

 

**Table 1:** Studies showing the association of types of anticoagulation with the outcomes

Serial No.	Study type	No.of Patients	Type of Anticoagulation	Amputation rate/limb salvage rate	Year of study
1.	Case Report by Goerlich et al.	Case report	Single antiplatelet therapy	Treated	2019
2.	Musonza et al.	Case report	Systemic anticoagulation	Treated	2019
3.	Retrospective study by Kim et al.	24 cases	Systemic anticoagulation	20.8%/Not stated	2019
4.	Retrospective study by Lebowitz et al.		Debatable	Variable	2018
5.	Retrospective study by Loja et al.	193 cases	Systemic anticoagulation	No decrease in amputation rates/increased need for blood products	2017
6.	Retrospective study by Maher et al.	323 cases	Systemic anticoagulation	Use associated with improved patency rate/no bleeding episode	2017
7.	Retrospective study by Liang et al.	-	Used regional/systemic anticoagulation	Outcome unclear	2016
8.	Retrospective study by Wang et al.	1524 cases	Systemic anticoagulation vs no anticoagulation	No difference in amputation rates	2016
9.	Humphries et al.		Systemic anticoagulation	Neither affected rate of limb loss nor side effects	2016
10.	Retrospective study by Hornez et al.		Regional hepsal solution		2015
11.	Retrospective study by Fahad et al.	328 cases	DVT prophylaxis with LMWH postoperatively	Not stated/41%	2015
12.	Retrospective study by Lang et al.	64 cases	Regional anticoagulation	28%/Not stated	2015
13.	Retrospective study by Klocker et al.	152 cases	Systemic anticoagulation	9.9%/Not stated	2014
14.	Retrospective study by di Silva et al.	70 cases	Regional anticoagulation	Not stated/92%	2011
15.	Retrospective review by Franz et al.	65 cases	Antiplatelet therapy in nonocclusive injuries	No amputation in this group	2011
16.	Retrospective study by J Klocker et al.	89 cases	Systemic anticoagulation	Not stated/98%	2010
17.	Retrospective study by Ali Pourzand et al.	62 cases	Regional anticoagulation	37%/Not stated	2010
18.	Woodward et al.	488 cases	regional	7.7%/Not stated	2008
19.	Retrospective study by Cakir et al.	192 cases	Systemic anticoagulation	variable	2005
20.	Retrospective study by Guerrero et al.	151 cases	Systemic anticoagulation	10.6%/Not stated	2002
21	Retrospective study by Hafez et al.	550 cases	Regional heparinized saline use	16.2%/84%	2001
22.	Retrospective study by Hunt et al.	Case report	Systemic anticoagulation	treated	2000
23.	Retrospective estudy by Eachempati et al.	23 cases	Systemic heparinization	Not stated	1998
24.	Retrospective study by Melton et al.	102 cases	Systemic anticoagulation	25%/Not stated	1997
25.	Retrospective study by Wagner et al.	109 Cases	Full dose Systemic heparinization	Not stated/85%	1994
26.	Retrospective study by Fitridge et al.	114 cases	Systemic intraoperative anticoagulation	23%/Not stated	1994
27.	Retrospective study by Daugherty et al.		Systemic anticoagulation	Not stated/91%	1978

Maher et al. conducted a retrospective cohort study of 323 patients from the multiple level I trauma centers, while Loja et al. conducted multilevel level I trauma centers (PROOVIT registry) prospective study on 193 patients [10,38]. Although Loja et al. described the use of systemic anticoagulation in extremity vascular trauma to be associated with an increase in the need for blood products and longer hospital stay [38]. The study does not show whether these patients had comorbids or multiple associated injuries that could have confounded the results of the study. As these patients already need more blood products or intensive care unit (ICU) stay. Furthermore, patients in this study had higher limb ischemia scores who received systemic anticoagulation than those who did not receive anticoagulation. So this might have contributed to increased use of blood products or hospital stay or limb amputation rates. Although many confounding factors like demographics, injury severity scores, Glasgow Coma Scale (GCS), or systolic blood pressure at admission were adjusted equally in both groups. Maher et al., at the same time, adjusted the confounding factors like concomitant injuries, heart rate, age, gender and mechanism of injury that could alter the results and showed improved patency rates in those received systemic intraoperative anticoagulation [10]. But this study included vascular injuries of torso, neck and proximal extremities that might have more dependence on anticoagulation to maintain the repair site patency while the smaller caliber vessels might not be affected by effects of anticoagulation.

 

Magnitude of the problem in terms of limb loss or life loss

Highest rates of limb loss are associated with popliteal artery trauma, which accounts for a 10% incidence in literature with more challenging management [14,21,40]. Inkellis et al. described amputation rates of 35% through the humerus, 30% at the forearm and 14% in hand [19]. Limb loss is significantly lower in upper limbs as compared to lower limbs [19,20]. Klocker et al. reported blunt trauma as a major cause of injury and the popliteal artery being the most common injury in the lower limb [20]. Limb survival rates are 95.6% to 96.2% and patient survival in 98.5% and primary amputation rate was 4.6% in extremely mangled limbs, fixed staining, gangrene, nonviable superficial posterior compartment with one other compartment with most amputations being above knee amputations (AKAs) and secondary amputation rate being 4.8% and 4.5% to 5.2% mostly due to occluded graft [14,16,18]. Graft thrombosis was 6.4% according to Khan et al. and 9.9% according to Klocker et al. Amputation rates are also described to be 10.5% and wound infection 13.1% [11,20]. Limb salvage rates of 41% are associated with the late presentation being after eight hours and more in lower limbs [11]. Lower extremity arterial injury is rising [18]. Guerrero et al. suggested the use of anticoagulation to be associated with reduced limb loss [35]. Amputation is caused most by firearm injuries and least by stab injury and the most common cause was failed revascularization in terms of occluded graft followed by combined injuries then tense compartments at presentation and then arterial transection and associated compound fractures [18]. Multidisciplinary team (MDT) was involved to address the associated injuries in addition to vascular injuries [14]. Loja et al. described the amputation rate of 11% [38].

Limb salvage rate following extremity vascular trauma is variable depending on the severity of tissue damage, mechanism of injury, time elapse in seeking emergency care, underlying hemodynamic status of the patient and associated concomitant injuries and use of anticoagulation in the perioperative period. Guerrero et al in a retrospective study of 151 patients described the use of perioperative anticoagulation to be associated with the lower limb loss (3.3%) as compared to those who did not receive it (15%) [35]. But those who received perioperative anticoagulation had lower injury severity scores than those who did not receive the anticoagulation because of the fear of bleeding complications from associated injuries. The mortality rate was 6.6%. Loja et al. in a retrospective study of 193 from PROOVIT registry described the amputation rate and repair thrombosis to be 11% without significant difference between those received intraoperative anticoagulation and those who did not (p-value 0.6) [38]. There were no deaths in this cohort. Besides, there was an increase in the use of blood products in those received anticoagulation. But this study does not provide ample information on the local anticoagulation use, antiplatelet therapy or any other forms of anticoagulation other than intraoperative systemic anticoagulation that could significantly alter the results. Strict contraindications to anticoagulation are not defined in these studies as well as the experience of the surgeon. While Guerrero et al. have categorized the patients into subgroups as those received subcutaneous heparin, intravenous heparin, low molecular weight heparin and intravenous dextran but there was no relationship of a specific route of administration of heparin and type of anticoagulation with improved limb salvage rates [35]. Both studies show higher limb loss associated with the popliteal artery injuries and the development of compartment syndrome.

Management of concomitant venous injuries

Extremity venous injuries are often associated with arterial injuries and are challenging to the operating surgeons and there is often debate about the repair vs ligation. Concomitant venous injuries are seen variably as 26%, 12.5%, and 22.4% in various studies [18,20]. These injuries are either ligated or grafted with vein or PTFE graft [14]. Venous injury treatment being simple lateral suturing or end-to-end anastomosis to save time and rest were ligated with no worse outcome and repair failure led to pulmonary embolism (PE) in 22% [18,41]. Venous repairs improve outcomes whenever possible to do and to do it before arterial repair to increase drainage of the limb [8,42]. But vein repair increases DVT risk so heparin to warfarin prophylaxis is given for three months [14]. Vein repair was done for popliteal vein, femoral vein and subclavian vein and rest ligated and primary repair of nerve injury was done in 59.7% and rest were tagged [11]. According to a retrospective study of 158 patients, the risk of PE following repair vs ligation is comparable [43]. Infrapopliteal repair of venous injuries has poor outcomes and is not necessary [44]. Prolonged anticoagulation after traumatic venous injury repair is not necessary [45]. The use of anticoagulation during microvascular anastomosis yields good better patency rates [46].

Concomitant venous injuries can be managed by either ligation or repair depending on the hemodynamic stability of the patients. Patients with venous repair are at risk of repair site thrombosis, DVT or PE. Some studies suggest that long-term anticoagulation beyond three days is not necessary to prevent these complications. Franz et al., in a retrospective review article, studied 66 patients with lower limb vascular injury patients suggested the use of venous repair over ligation whenever possible to increase the drainage of the limb but long-term DVT thromboprophylaxis was given for three months after repair [14]. Allen et al. described that risk of PE following repair vs ligation is comparable so there is no need for long-term anticoagulation [43]. Moreover, patients who developed a PE in this study were already on thromboprophylaxis. The sample size of review of Franz et al. was 66 patients, while Allen et al. reviewed data from 158 patients but this study included only patients with penetrating trauma that might have lesser effects on the development of PE [14,43].

 

Limitations

This review article is based on the studies, which are mostly retrospective studies and case reports. Limb salvage rates and mortality rates are variable among the studies because of variable management strategies. There is no clear cut indication identifiable as when to use systemic anticoagulation and when to use regional anticoagulation and antiplatelet therapy, it was variable according to each piece of literature we could find. There are various confounding factors that can affect the amputation rates and outcomes. Current literature has scarce follow-up of patients in terms of long-term morbidities in surviving limbs as some nonvascular morbidities may lead to amputation later and could provide important retrospective information about the management strategies of traumatic vascular injuries and this information could be used in future patients.

## Conclusions

Extremity vascular trauma is a vivid domain of emergency surgery, relying on vascular surgeons and trauma surgeons. Vascular injuries do not give a wide margin of time to the healthcare staff to take measures to save the limb or to save a life. The use of intraoperative anticoagulation at the time of vascular repair is an important determinant in terms of repair site thrombosis or limb loss. Its role is well established in the elective vascular repairs but in emergency cases, it is still an unresolved riddle. In some studies, the use of intraoperative anticoagulation is related to increased blood loss. Most of the studies demonstrate that the use of anticoagulation during extremity vascular trauma is associated with improved limb salvage rates but more comprehensive studies and randomized controlled trials are needed to confirm the importance of perioperative anticoagulation while avoiding the confounding factors in terms of injury severity scores, ischemia time, demographics of patients, modes of injury, comorbidities, grades of shock, concomitant injuries that need anticoagulation like venous injuries or intracranial injuries that are contraindications to the use of anticoagulation, type of anticoagulation and expertise available as well as the experience level of the operating surgeon. Role of antiplatelets vs anticoagulants needs to be addressed but this may be outside the domain of this article.

Literature also reveals the use of new oral anticoagulants (e.g., dabigatran) to be associated with lesser bleeding episodes so in the future we can check the feasibility of these agents to reduce the bleeding episodes and at the same time improve the limb salvage rates. Besides the disadvantage of anticoagulation in terms of bleeding episodes may be used as a benefit in terms of permissive hypotension, required in many trauma patients in case if it is confirmed that anticoagulation reduces repair site thrombosis by randomized controlled trials. Long-term follow-up is required. The importance of anticoagulation lies in the fact that limb loss is a major life long morbidity for the patient, for the family, and for the communities at the same time. We should make every effort to avoid it by providing best possible care and modifying those prognostic factors which are in the hand of a surgeon and which could result in saving a limb, saving self-esteem, saving confidence and saving a smile or on the contrary leaving a patient into the darkness of life long misery.
